# Engineering of *Escherichia coli* to facilitate efficient utilization of isomaltose and panose in industrial glucose feedstock

**DOI:** 10.1007/s00253-016-8037-z

**Published:** 2016-12-08

**Authors:** Kenji Abe, Akio Kuroda, Ryo Takeshita

**Affiliations:** 1 Process Development Laboratories, Research Institute for Bioscience Products & Fine Chemicals, Ajinomoto Co., Inc., 1-1 Suzuki-cho, Kawasaki-ku, Kawasaki-shi, Kanagawa 210-8681 Japan; 2 Department of Molecular Biotechnology, Graduate School of Advanced Sciences of Matter, Hiroshima University, 1-3-1 Kagamiyama, Higashi-Hiroshima-shi, Hiroshima, 739-8530 Japan

**Keywords:** *Escherichia coli*, Maltose, Isomaltose, Panose, Assimilation, *glvA*, *glvC*, *Bacillus subtilis*, l-Lysine production

## Abstract

Industrial glucose feedstock prepared by enzymatic digestion of starch typically contains significant amounts of disaccharides such as maltose and isomaltose and trisaccharides such as maltotriose and panose. Maltose and maltosaccharides can be utilized in *Escherichia coli* fermentation using industrial glucose feedstock because there is an intrinsic assimilation pathway for these sugars. However, saccharides that contain α-1,6 bonds, such as isomaltose and panose, are still present after fermentation because there is no metabolic pathway for these sugars. To facilitate more efficient utilization of glucose feedstock, we introduced *glvA*, which encodes phospho-α-glucosidase, and *glvC*, which encodes a subunit of the phosphoenolpyruvate-dependent maltose phosphotransferase system (PTS) of *Bacillus subtilis*, into *E. coli.* The heterologous expression of *glvA* and *glvC* conferred upon the recombinant the ability to assimilate isomaltose and panose. The recombinant *E. coli* assimilated not only other disaccharides but also trisaccharides, including alcohol forms of these saccharides, such as isomaltitol. To the best of our knowledge, this is the first report to show the involvement of the microbial PTS in the assimilation of trisaccharides. Furthermore, we demonstrated that an l-lysine-producing *E. coli* harboring *glvA* and *glvC* converted isomaltose and panose to l-lysine efficiently. These findings are expected to be beneficial for industrial fermentation.

## Introduction

In the industrial production of useful compounds by fermentation, glucose is one of the most frequently used carbon sources (Peters 2007). Industrial glucose feedstock is prepared from starch, a polysaccharide composed of glucose units linked together by α-1,4 and α-1,6 glycoside bonds, by means of enzymatic hydrolysis (Martin and Smith 1995; Hii et al. 2012). Complete hydrolysis of starch into glucose adds significant cost; therefore, most commercially available glucose feedstock is processed incompletely (Gokarn et al. 2014). Because of incomplete enzymatic hydrolysis and/or reverse reactions, the glucose feedstock contains significant amounts of maltose [4-O-α-D-glucopyranosyl-D-glucopyranose] (1–2%), isomaltose [6-O-α-D-glucopyranosyl-D-glucopyranose] (0.5–2%), and other oligosaccharides, such as panose [α-D-glucopyranosyl-(1->6)-α-D-glucopyranosyl-(1->4)-D-glucopyranose] (1% or less) (Hii et al. 2012; Gokarn et al. 2014; Hassan et al. 1998; Crabb and Shetty 1999; Sierkes and Svensson 1992; Takasaki 1988; Chaplin and Bucke 1994). If microorganisms used for fermentation cannot metabolize these saccharides, valuable carbohydrates would be wasted.

*Escherichia coli* is the most useful bacterial strain for the production of valuable compounds, such as amino acids and organic acids, because *E. coli* cells grow quickly, rapidly convert substrates to products, and are readily genetically engineered (Leuchtenberger et al. 2005; Wendisch et al. 2006). For example, l-lysine, which is used as a feed additive worldwide, is produced on the scale of approximately 1,500,000 t per year (Doi et al. 2014). However, saccharides that contain α-1,6 bonds, such as isomaltose and panose, are not used up during *E. coli* fermentation because *E. coli* cannot assimilate isomaltose and panose as carbon sources. Furthermore, these sugars, which contain reducing sugar moieties, can react with free amino groups of amino acids during the purification step (Smuda and Glomb 2011; Ledl and Schleicher 1990); this so-called Maillard reaction decreases the yield of the final product and contaminates the reaction mixture with undesirable compounds. These problems must be overcome in order to increase the yield and productivity of fermentation when using glucose feedstock as a carbon source.

The phosphotransferase system (PTS) is responsible for the transport and phosphorylation of sugars. The multi-component PTS comprises a phosphohistidine carrier protein (HPr), an enzyme I (EI) component, and a membrane-bound enzyme complex (EII). The HPr and EI components transfer a phosphoryl group of phosphoenolpyruvate (PEP) to the sugar-specific enzymes EIIA and EIIB. EIIC is an integral membrane protein permease that recognizes and transports the sugar, which is then phosphorylated by EIIB (Postma et al. 1993). There are 21 different EII complexes encoded in the *E. coli* chromosome; these complexes are involved in the transport of approximately 20 different carbohydrates (Escalante et al. 2012). Pikis et al. reported that the heterologous expression of *Klebsiella pneumoniae aglA* (a single-chain polypeptide of EIIC and EIIB that mediates the transport and phosphorylation of sucrose and various other α-linked glucosides) and *aglB* (a phospho-α-glucosidase) confers upon *E. coli* cells the ability to utilize isomaltose (Pikis et al. 2006; Thompson et al. 2001). Although *E. coli* K-12 strains have homologs of *aglA* and *aglB* (Thompson et al. 2001), these seemed to be cryptic or nonfunctional truncated proteins (Reizer 1994; Thompson et al. 1995). However, *Bacillus subtilis* strains, which are generally regarded as safe (GRAS) organisms by the Food and Drug Administration (FDA) (Harwood and Wipat 1996; Singh et al. 2009; Song et al. 2016; Zeigler et al. 2008), have *glvA* and *glvC*, functional homologs of *aglB* and *aglA*, respectively (Thompson et al. 2001). GlvA and GlvC are known to be involved in maltose assimilation in *B. subtilis* (Yamamoto et al. 2001; Thompson et al. 1998). Although a wide variety of phosphorylated α-linked aryl glucosides can be degraded by GlvA (Yip et al. 2007), there are no other reports describing its substrate specificity.

In this study, we found that the heterologous expression of *glvA* and *glvC* conferred upon *E. coli* cells the ability to assimilate isomaltose. Unexpectedly, the recombinant also assimilated trisaccharides containing α-1,6 bonds, such as panose, as well as the alcohol forms of these saccharides, such as isomaltitol. Our results may facilitate increased production yields using glucose feedstock in industrial-scale fermentation by *E. coli*.

## Materials and methods

### Bacterial strains, plasmids, and primers

All strains, plasmids, and primers used in this study are listed in Tables 1 and 2.

**Table 1:** Strains and plasmids used in this study

Strain or plasmid	Description, genotype, or sequence	Reference, source
Strains		
*E. coli* K-12 MG1655	F^−^ l^−^ *ilvG rfb*-*50 rph*-*1*	CGSC Collection
*E. coli* WC196LC	W3110 NTG mutant (S-aminoethyl-L-cysteine-resistant mutant) Δ*ldc* Δ*cadA*	Kikuchi et al. (1997)
*Bacillus subtilis* 168	*trpC2 ypqP*::SPβ	Zeigler et al. (2008)
MG1655 (empty vector)	*E. coli* K-12 MG1655 harboring pTWV229 and pMW219-Δplac	This study
MG1655 (glvAC)	*E. coli* K-12 MG1655 harboring pTWV229-self-glvA-Fw and pMW219-ΔPlac-*tac-glvC	This study
WC196LC (pCABD2)	*E. coli* WC196LC harboring pCABD2	Kojima et al. (1994); Kikuchi et al. (1997); Doi et al. (2014)
Plasmids		
pTWV229	Cloning vector, Ap^r^	Takara Bio Inc., (Japan)
pMW219	Cloning vector, Km^r^	Nippon Gene Co. Ltd. (Japan)
pMW219-ΔPlac	pMW219 derivative lacking the *lac* promoter region	This study
pMW219-ΔPlac-tac-glvC-R2	pMW219-ΔPlac derivative harboring the Ptac-*glvC* gene	This study
pMW219-ΔPlac-Ptac4075-glvC-Rv	pMW219-ΔPlac derivative harboring the Ptac4075-*glvC* gene	This study
pTWV229-self-glvA-Fw	pTWV229 derivative harboring the Pself-*glvA* gene	This study
pMW219-ΔPlac-*tac-glvC	pMW219-ΔPlac-tac-glvC-R2 derivative harboring a mutation in the −10 region of the tac promoter (TATAAT to AATAAT)	This study
pCABD2	pRSF1010 harboring mutated *lysC*, mutated *dapA*, mutated *dapB*, and *C. glutamicum ddh*	Kojima et al. (1994)

**Table 2:** Primers used in this study

Primer	Sequence
pMW119-F-Hind3	GCCAAGCTTGCATGCCTGCAGGTCGACTCTAGAGG
pMW119-R-Hind3	CCCAAGCTTGCTAACTCACATTAATTGCGTTG
pTWV229-F-Hind3	GCCAAGCTTGCATGCCTGCAGGTCGACTCTAGAGG
pTWV229-R-Hind3	CCCAAGCTTCACATTACTTGGCAGAACATATCC
glvC-F-tac	CAATTTCACACAAGGAGACTGCCATGATGCAAAAAATTCAGCG
glvC-R2	CCCAAGCTTCCCCTTTTTACTCGATTGTCTC
tac-promoter-glvC-1	CGTATAATGTGTGGAATCGTGAGCGGATAACAATTTCACACAAGGAGACTGCCATGATGCAAAAAATTCAGCGCTTTGGA
Hind3-tac-promoter	CCCAAGCTTCCTGTTGACAATTAATCATCGGCTCGTATAATGTGTGGAATCGTGAGCGGATAACAATTTCACACAAGGAG
tac-promoter-glvC-2	CGAATAATGTGTGGAATCGTGAGCGGATAACAATTTCACACAAGGAGACTGCCATGATGCAAAAAATTCAGCGCTTTGGA
glvA-self-Fw1	AGAAATTTCCCGCTCTATGG
glvA-self-Rv1	TGTAGTGCTGATTGATCAGTTC

HindIII recognition site was underlined

### Construction of vectors

The plasmid pMW219-ΔPlac was constructed by deleting the *lac* promoter from the vector plasmid pMW219 (Nippon Gene Co., Ltd., Tokyo, Japan) as follows. A DNA fragment was amplified using the primer set pMW119-F-Hind3 and pMW119-R-Hind3, and *Hin*dIII/*Pst*I-digested pMW219 was used as a template. The polymerase chain reaction (PCR)-amplified fragment was digested by *Hin*dIII and subsequently self-ligated by DNA ligase. *E. coli* JM109 competent cells were transformed with the DNA, and transformants were selected on LB agar medium containing kanamycin.

### Construction of *glvC*-expressing plasmids

A DNA fragment containing *glvC* was amplified by PCR with the primer set glvC-F-tac and glvC-R2 and with the *B. subtilis* 168 genome as a template. In order to add a promoter sequence upstream of *glvC*, the amplified DNA fragment containing *glvC* and synthetic single-strand DNA (tac-promoter-glvC-1) were mixed, and another PCR was then carried out using the primer set Hind3-tac-promoter, glvC-R2. The amplified DNA fragment and *Sma*I-digested pMW219-ΔPlac were ligated by DNA ligase. The plasmid pMW219-ΔPlac-glvC-R2 was extracted from transformants, and its structure was confirmed. The plasmid pMW219-ΔPlac-Ptac4075-glvC-Rv containing *glvC* under the control of the *tac* promoter variant Ptac4075, which was a weaker promoter than *tac* promoter because of a mutation in the consensus sequence, was constructed in a similar manner using primers Hind3-tac-promoter, glvC-R2, and synthetic DNA, tac-promoter-glvC-2.

### Construction of *glvA*-expressing plasmid

A DNA fragment containing *glvA* and its upstream region containing a promoter sequence was amplified by PCR with the primer set glvA-self-Fw1 and glvA-self-Rv1 and with the *B. subtilis* 168 genome as a template. The PCR-amplified DNA and *Sma*I-digested pTWV229 were ligated by DNA ligase. In the resulting plasmid, pTWV229-self-glvA-Fw, *glvA* mRNA was transcribed via the *lac* promoter of pTWV229.

### Assimilation test in M9 minimal medium

M9 liquid minimal medium (Miller 1992) supplemented with 2 g/L isomaltose or maltose was used for assimilation tests. *E. coli* strains were precultured overnight at 37 °C on LB medium. The cells were washed three times with cold saline and adjusted to an OD_620_ of 7.0. The cell suspension (70 μL) was added to 5 mL M9 minimal medium in an L-shaped test tube and cultured at 37 °C with shaking at 70 rpm using a Bio-Photorecorder (TN-1506; Advantec, Inc., Tokyo, Japan). In all experiments, appropriate antibiotics were added to the medium. M9 solid minimal medium (Miller 1992) supplemented with 2 g/L of various types of sugars and sugar alcohols was used for assimilation tests. *E. coli* strains were precultured overnight at 37 °C on LB medium. The cells were washed three times by cold saline and adjusted to OD_620_ of 5.0. The cell suspension (20 μL) was inoculated on M9 minimal medium plates containing various types of sugars and sugar alcohols and incubated at 37 °C for 48 h. Glucose and sucrose were purchased from Junsei Chemical Co., Ltd. (Tokyo, Japan). Maltose was purchased from Nacalai Tesque, Inc. (Kyoto, Japan). α-Methyl-glucoside was purchased from Wako Pure Chemical Industries, Ltd. (Osaka, Japan). Isomaltulose, maltotriitol, isomaltitol, lactitol, and erlose were purchased from Hayashibara Co., Ltd. (Okayama, Japan). Isomaltose, panose, isomaltotriose, maltitol, trehalose, turanose, maltulose, galactinol, cellobiose, gentiobiose, lactose, melibiose, lactulose, maltotriose, maltotetraose, maltopentaose, and raffinose were purchased from Tokyo Chemical Industry Co., Ltd. (Tokyo, Japan). For solid medium, 15 g/L Bacto agar (Becton Dickinson and Company, USA) was added.

### Assimilation tests for maltose and isomaltose in the presence of glucose

*E. coli* MG1655 harboring pTWV229-self-glvA-Fw and pMW219-ΔPlac-*tac-glvC (obtained by an unintended mutation, as described in the “Results” section) was inoculated into M9 minimal medium containing 1.0 g/L maltose or isomaltose combined with 1.0 g/L glucose. Cells were then cultured at 37 °C with shaking at 70 rpm using a Bio-Photorecorder (Advantec), and sugar concentrations were assayed.

### l-Lysine production using glucose, maltose, isomaltose, and panose as carbon sources

The l-lysine-producing strain WC196LC harboring pCABD2 [encoding *dapA24*, *lysC80*, *dapB*, and *ddh* (Kojima et al. 1994; Kikuchi et al. 1997; Doi et al. 2014)] was transformed with pMW219-ΔPlac-Ptac4075-glvC and pTWV229-self-glvA-Fw. The transformant was inoculated on an LB plate containing 20 mg/L streptomycin, 100 mg/L ampicillin, and 50 mg/L kanamycin and incubated at 37 °C for 24 h. Colonies were scratched off, suspended in saline, and adjusted to an OD_620_ of 15. Next, 250 μL of the cell suspension was added to 5 mL L-lysine production medium containing 16 g/L glucose, 1.6 g/L maltose and/or isomaltose and/or 1.6 g/L panose, 1 g/L MgSO_4_ heptahydrate, 24 g/L (NH_4_)_2_SO_4_, 1 g/L KH_2_PO_4_, 2 g/L yeast extract, 0.1 g/L isoleucine, 12 mg/L FeSO_4_ heptahydrate, 9.6 mg/L MnSO_4_ pentahydrate, 30 g/L CaCO_3_ (as dry heat-sterilized powder), 20 mg/L streptomycin, 100 mg/L ampicillin, and 50 mg/L kanamycin (pH 7.0 with KOH). Cells were then cultivated at 37 °C for 41 h with reciprocal shaking at 120 rpm. Glucose and l-lysine were assayed by a biotech analyzer (AS310; Sakura Si Co., Ltd., Tokyo, Japan). Maltose, isomaltose, and panose were measured using an ICS-3000 Ion Chromatography System with a CarboPac PA1 column (Dionex, CA, USA).

## Results

### Evaluation of the functions of GlvA and GlvC from *B. subtilis* in *E. coli*

**Fig. 1:**
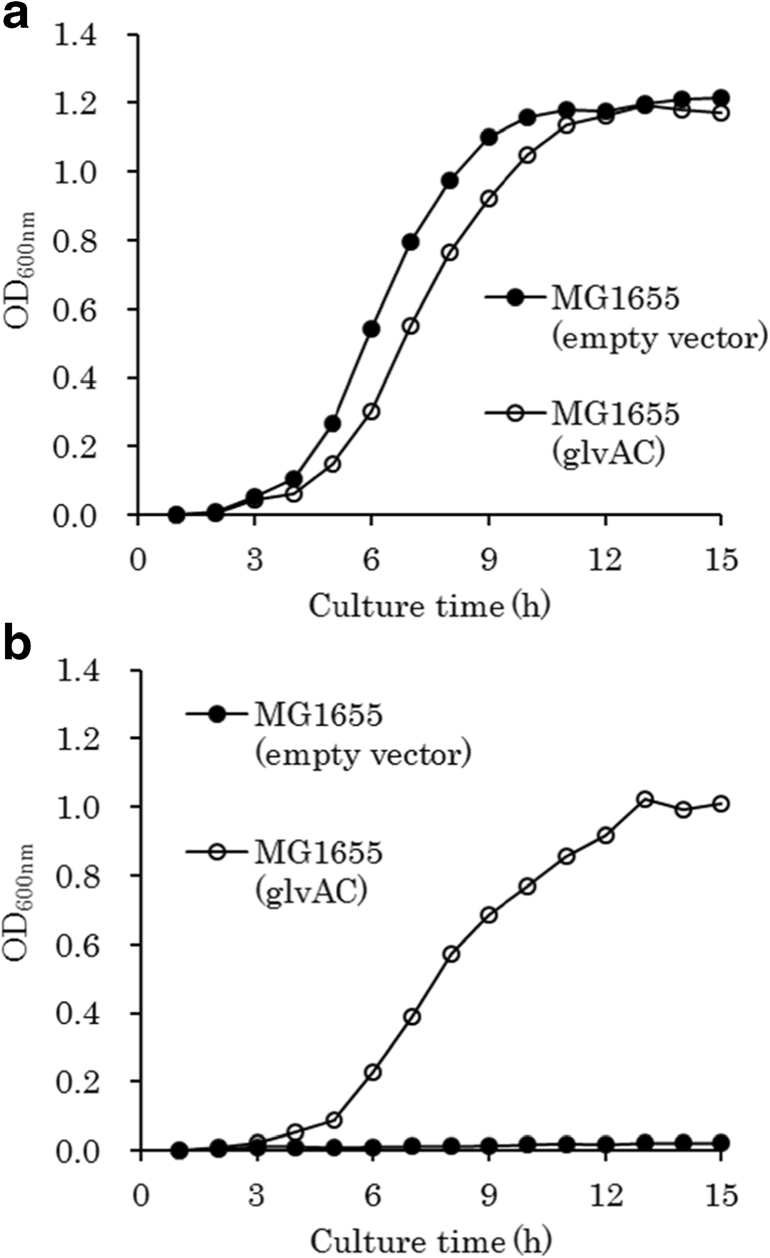
Growth curves of *E. coli* strains on M9-glucose (**a**) and M9-isomaltose (**b**). Each point in the curve represents the mean of two independent experiments

GlvA and GlvC have been reported to be involved in the assimilation of maltose in *B. subtilis* (Yamamoto et al. 2001; Thompson et al. 1998)*.* We constructed the plasmids pTWV229-self-glvA-Fw and pMW219-ΔPlac-tac-glvC-R2 for the expression of *glvA* and *glvC* from *B. subtilis* in *E. coli*. The growth of the *E. coli* MG1655 recombinant harboring both of the plasmids on M9 medium containing isomaltose was severely limited (data not shown). However, after prolonged incubation (45 h) of the recombinant at 37 °C on the isomaltose medium, we found that some mutants started to form larger colonies. Because plasmids isolated from one of the mutants enabled *E. coli* to grow rapidly on medium containing isomaltose, we analyzed the DNA sequences of these plasmids. A mutation was found in the −10 region of the *tac* promoter (de Boer et al. 1983) (TATAAT to AATAAT) upstream of *glvC*. This mutation is expected to reduce the expression level of *glvC* by affecting the binding affinity of RNA polymerase. We speculated that strong expression of GlvC, a membrane permease, may be toxic in *E. coli*. The mutant plasmid was renamed pMW219-ΔPlac-*tac-glvC. Next, we tested the growth of *E. coli* MG1655 harboring pTWV229-self-glvA-Fw and pMW219-ΔPlac-*tac-glvC plasmids in M9 liquid medium containing isomaltose as a sole carbon source. The recombinant could grow on isomaltose efficiently, whereas *E. coli* harboring the empty vector plasmids could grow only on glucose (Fig. 1).

### Simultaneous assimilation of glucose and isomaltose by *glvA*-expressing and *glvC*-expressing *E. coli*

**Fig. 2:**
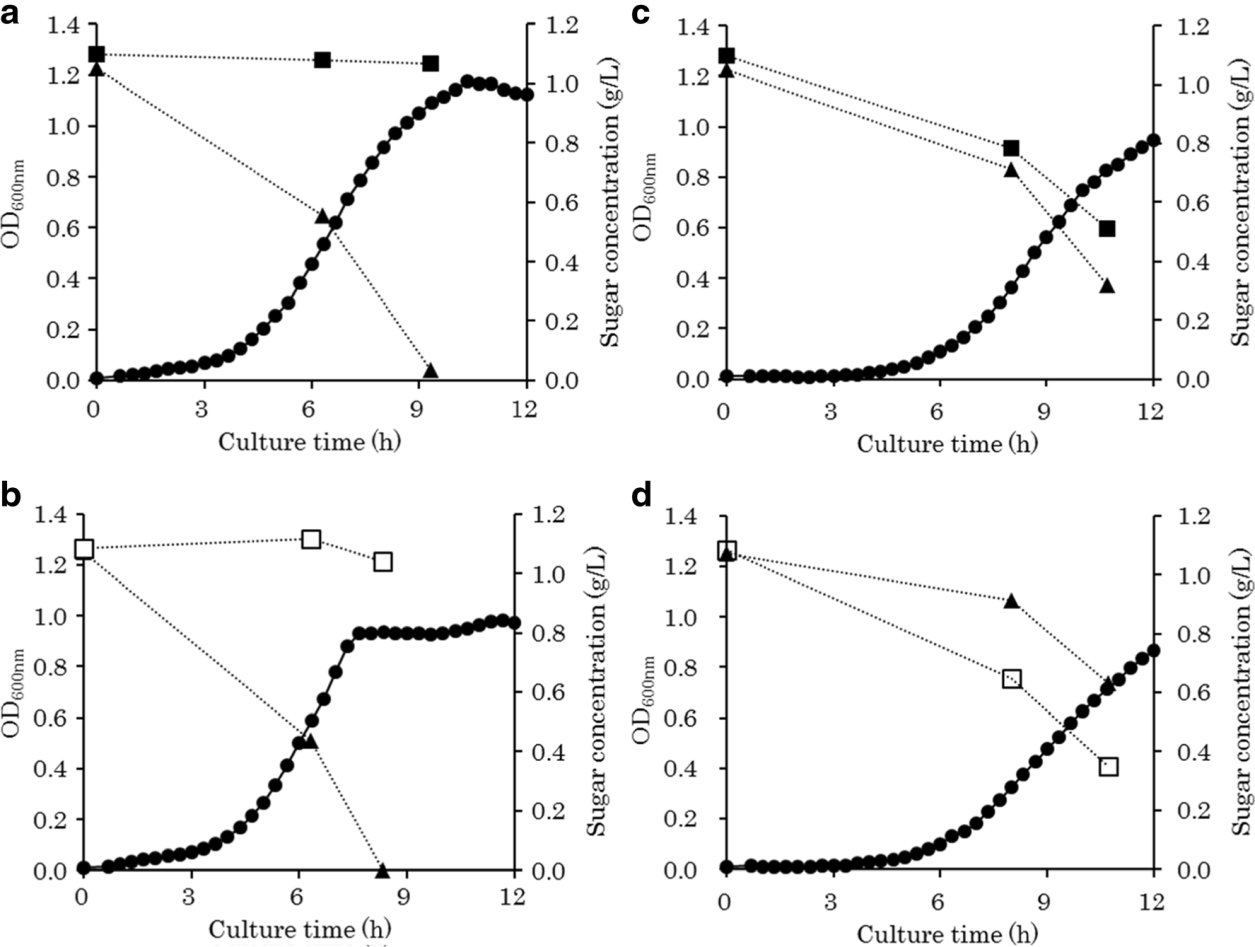
Growth curves of *E. coli* recombinants on M9-glucose-maltose or M9-glucose-isomaltose. **a** MG1655 (empty vector) on M9-glucose-maltose. **b** MG1655 (empty vector) on M9-glucose-isomaltose. **c** MG1655 (glvAC) on M9-glucose-maltose. **d** MG1655 (glvAC) on M9-glucose-isomaltose. Cell growth (*closed circle*), glucose concentration (*closed triangle*), maltose concentration (*closed square*), isomaltose concentration (*open square*)

To test whether the *E. coli* recombinant could assimilate isomaltose without catabolite repression, we used M9 medium containing an excess amount (1.8 g/L) of isomaltose or maltose supplemented with a small amount (0.2 g/L) of glucose (Loomis and Magasanik 1967). In this medium, cell growth would stop temporarily at an OD_600_ of 0.1–0.2 if the strain consumed glucose first (showing diauxic growth). The *E. coli* recombinant showed relatively slow but smooth cell growth, without characteristics of diauxic growth (data not shown), suggesting that the *E. coli* recombinant could assimilate isomaltose without catabolite repression. Then, to demonstrate this phenomenon more clearly, we measured sugar concentrations during cultivation on M9 minimal medium containing 1.0 g/L maltose and glucose or 1.0 g/L isomaltose and glucose. The *E. coli* carrying empty vector did not assimilate maltose or isomaltose in the presence of glucose (Fig. 2a, b), because *E. coli* assimilates maltose under the control of catabolite repression (Dean et al. 1990; Boos and Shuman 1998) and cannot assimilate isomaltose. On the other hand, the recombinant assimilated maltose or isomaltose in the presence of glucose (Fig. 2c, d). Surprisingly, the recombinant preferentially assimilated isomaltose over glucose (Fig. 2d). These results indicated that the heterologous expression of *glvA* and *glvC* conferred upon the cells the ability to assimilate maltose and isomaltose even in the presence of glucose.

### Evaluation of the substrate specificity of GlvA and GlvC in *E. coli*

**Fig. 3:**
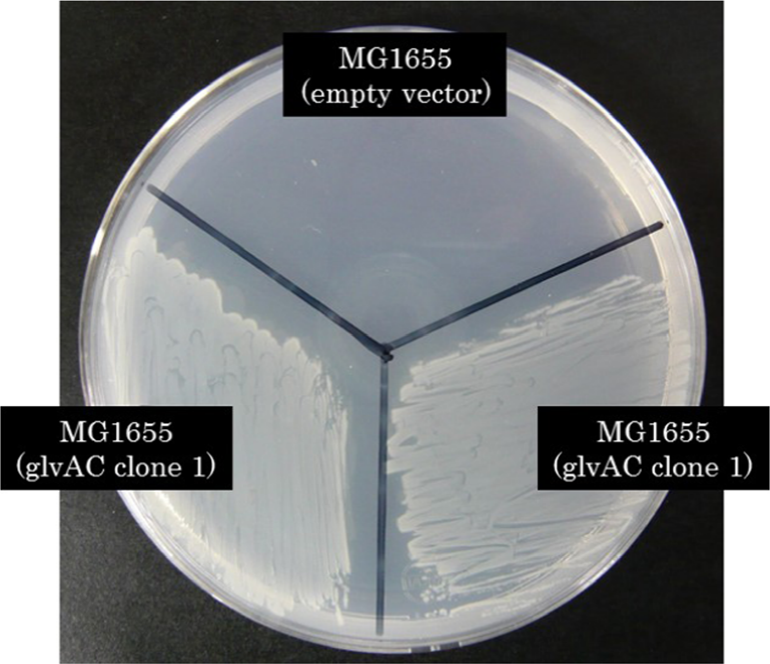
Growth of *E. coli* recombinants on M9 solid medium containing panose as a sole carbon source. Two clones were tested in the case of MG1655 (glvAC)

Pikis et al. reported that that expression of *aglA* and *aglB* from *K. pneumoniae* in *E. coli* allowed the cells to assimilate α-methyl-glucoside, isomaltose, trehalulose, turanose, maltulose, leucrose, and isomaltulose (Pikis et al. 2006). To investigate whether the heterologous expression of *glvA* and *glvC* enabled *E. coli* to assimilate other types of carbon sources, particularly panose, we performed growth tests using M9 solid medium supplemented with various types of sugars and sugar alcohols as a sole carbon source. We tested glucose, α-methyl-glucoside, sucrose, maltose, isomaltose, maltitol, trehalose, maltulose, isomaltulose, galactinol, cellobiose, gentiobiose, lactose, melibiose, lactulose, maltotriose, panose, isomaltotriose, maltotetraose, raffinose, maltotriitol, isomaltitol, lactitol, erlose, and maltopentaose (Table 3 and Fig. 3). Our results showed that the recombinant could assimilate many types of sugars and sugar alcohols, including α-methyl-glucoside, isomaltose, turanose, maltulose, and isomaltulose, which had been reported to be assimilated by AglA and AglB (Pikis et al. 2006). In contrast, galactinol, maltotriitol, and erlose could not be assimilated by the recombinant. Interestingly, the recombinant was able to assimilate trisaccharides, such as panose and isomaltotriose. Moreover, β-linked disaccharides such as sucrose and gentiobiose were also assimilated by the recombinant. It is an unexpected result because GlvA is classified as a 6-phospho-α-glucosidase (Yip et al. 2007).

**Table 3:** Growth of *E. coli* recombinants on selected sugars

Substrate	DP	Form	MG1655 (empty vector)	MG1655 (glvAC)	MG1655 (pAP2) (Pikis et al. 2006)
Glucose	1	Sugar	++	++	++
α-Methyl-glucoside	1	Sugar	NDG	++	++
Sucrose	2	Sugar	NDG	++	−
Maltose	2	Sugar	++	++	++
Isomaltose	2	Sugar	NDG	++	++
Maltitol	2	Sugar alcohol	NDG	++	++
Trehalose	2	Sugar	++	++	++
Turanose	2	Sugar	+	++	++
Maltulose	2	Sugar	NDG	++	++
Isomaltulose	2	Sugar	NDG	++	++
Galactinol	2	Sugar alcohol	NDG	NDG	No information
Cellobiose	2	Sugar	+	+	No information
Gentiobiose	2	Sugar	NDG	+	No information
Lactose	2	Sugar	++	++	No information
Melibiose	2	Sugar	++	++	No information
Lactulose	2	Sugar	++	++	No information
Maltotriose	3	Sugar	++	++	No information
Panose	3	Sugar	NDG	++	No information
Isomaltotriose	3	Sugar	NDG	++	No information
Maltotetraose	4	Sugar	++	++	No information
Raffinose	3	Sugar	+	++	No information
Maltotriitol	3	Sugar alcohol	NDG	NDG	No information
Isomaltitol	3	Sugar alcohol	NDG	++	No information
Lactitol	2	Sugar alcohol	++	++	No information
Erlose	3	Sugar	NDG	NDG	No information
Maltopentaose	5	Sugar	++	++	No information

Data of MG1655 (pAP2) which expresses *aglA* and *aglB* from *K. pneumoniae* are described in the reference (Pikis et al. 2006) and listed in this table for comparison to show what kinds of sugars and sugar alcohols were newly assimilated by heterologous expression of *glvA* and *glvC*

*DP* degree of polymerization, *NDG* no detectable growth, − minimal growth, + slight growth, ++ clear growth

The expression of either *glvA* or *glvC* alone is not sufficient to confer upon the recombinant the ability to assimilate any of the tested sugars and sugar alcohols except α-methyl-glucoside. It is known that α-methyl-glucoside is transported by PtsG of *E. coli* with concomitant phosphorylation (Pikis et al. 2006). GlvA can hydrolyze the phosphorylated α-methyl-glucoside, conferring the ability to grow on the medium containing α-methyl-glucoside.

A common chemical structure among the substrates assimilated by the GlvA-expressing and GlvC-expressing recombinant was the presence of glucose at one terminal of the sugar or sugar alcohol. Therefore, GlvC appeared to recognize the glucose unit of the sugar or sugar alcohol and transport the unit with concomitant phosphorylation of the glucose terminal. Moreover, GlvA may hydrolyze the 6′-phospho-sugars and sugar alcohols to release glucose 6-phosphate. This is the first report to show expansion of the sugar substrates of *E. coli* to trisaccharides by heterologous expression of 6-phospho-α-glucosidase and PTS components.

### Utilization of isomaltose and panose using GlvA and GlvC in an l-lysine-producing model strain

**Fig. 4:**
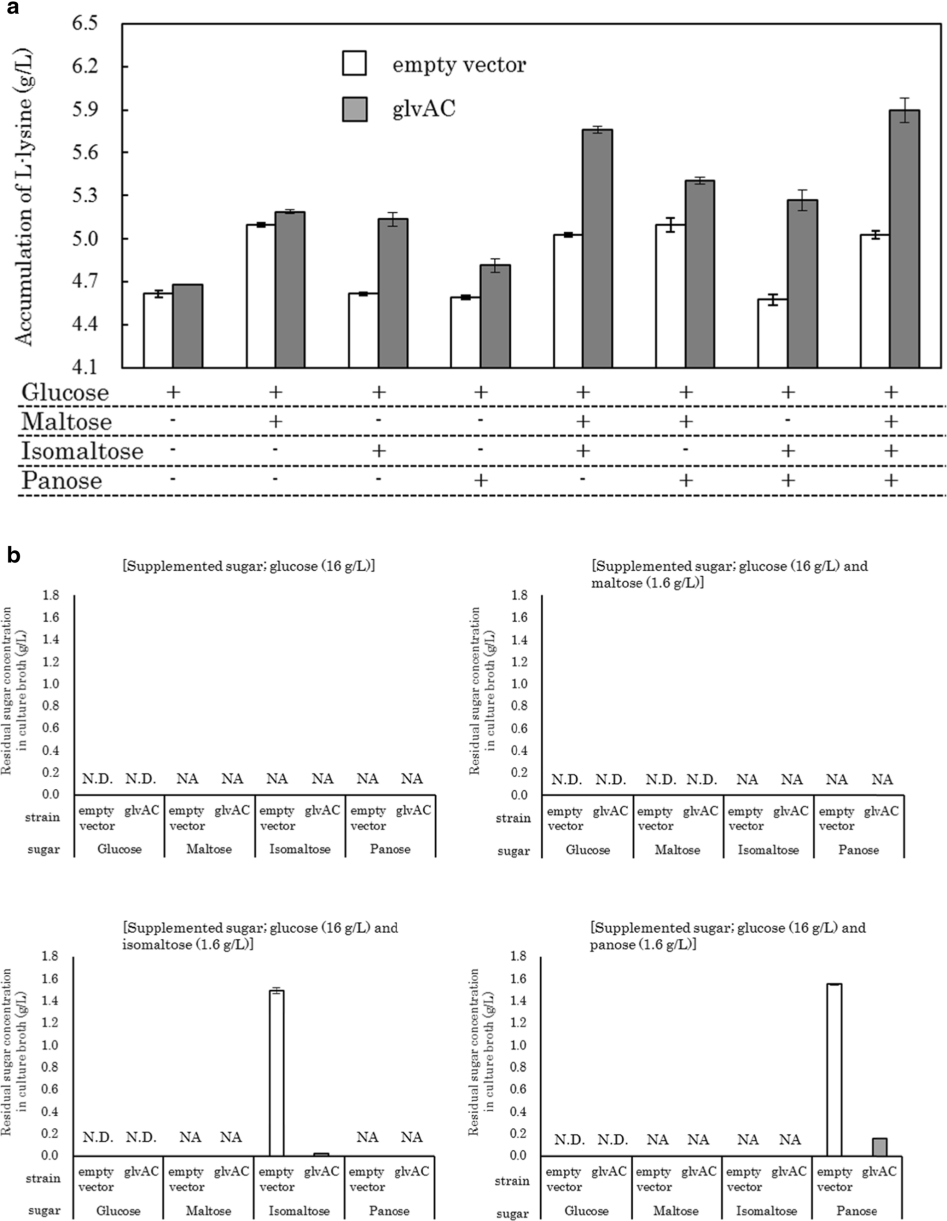

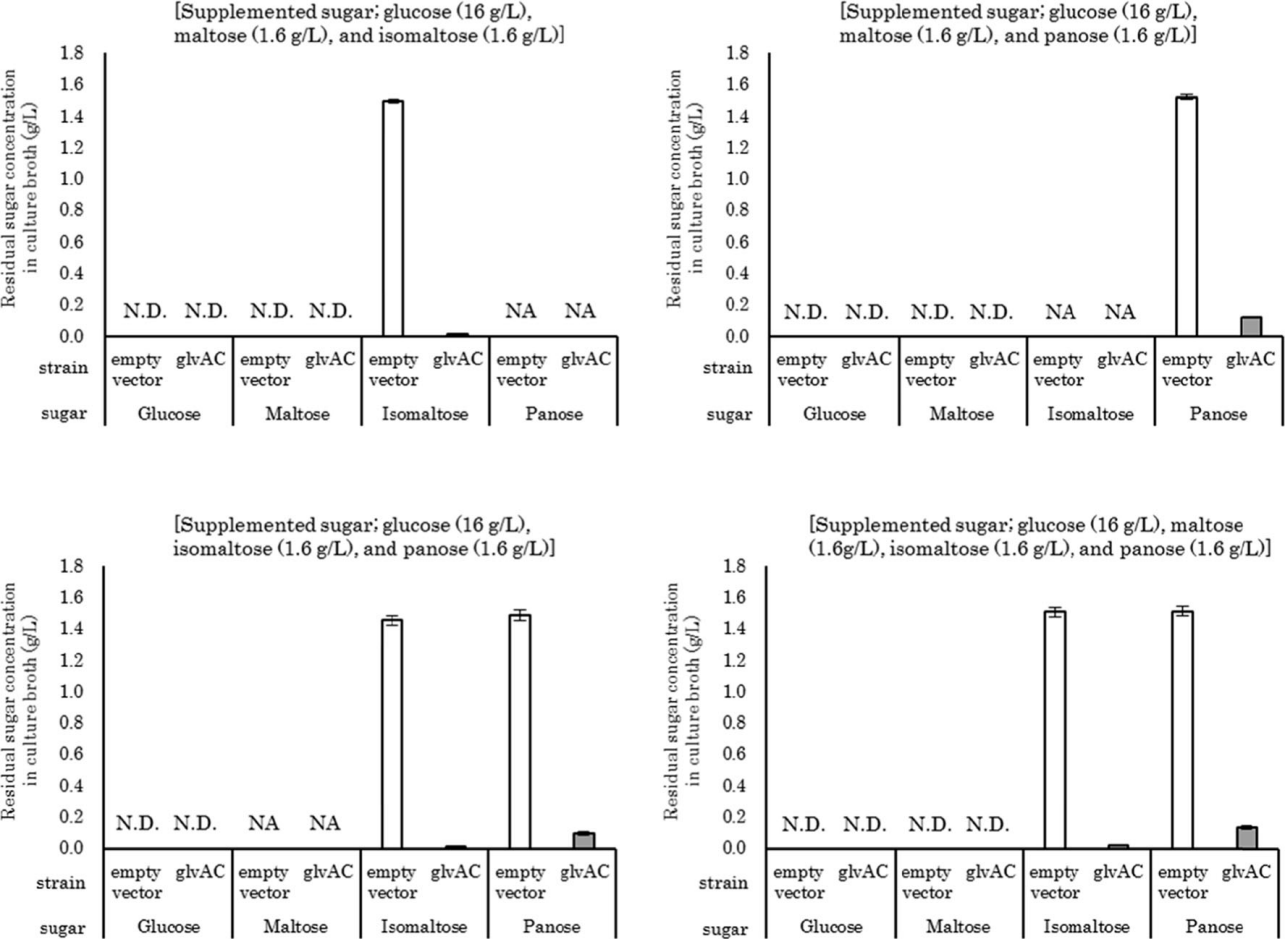
Utilization of maltose, isomaltose, and panose in the l-lysine-producing model strain. **a** Accumulation of l-lysine in the l-lysine production medium supplemented with glucose or glucose combined with maltose, isomaltose, and panose at the end of fermentation. **b** Residual maltose, isomaltose, and panose in the culture broth at the end of fermentation. Values are the means of more than three independent samples. *SE bars* represent the standard error of the mean calculated with Excel software. WC196LC (pCABD2) harboring the empty vector plasmids, pTVW229 and pMW219-Δplac (*empty vector*); WC196LC (pCABD2) harboring the *glvA*-expressing and *glvC*-expressing plasmids, pTWV229-self-glvA-Fw and pMW219-ΔPlac-Ptac4075-glvC (*glvAC*); *N.D.* not detected, *NA* no addition

To evaluate the effects of isomaltose and panose utilization on fermentation efficiency, we introduced the plasmids to an l-lysine-producing *E. coli* strain WC196LC (pCABD2) (Kojima et al. 1994; Kikuchi et al. 1997; Doi et al. 2014). The recombinant was cultivated on l-lysine production medium supplemented with glucose or glucose combined with isomaltose, panose, or maltose (as a control). Additionally, media containing different combinations of the above saccharides were also prepared and used for l-lysine production tests. l-Lysine accumulation in the culture broth of *E. coli* WC196LC (pCABD2) harboring the empty vector plasmids was increased by approximately 0.5 g/L only when maltose, which can be assimilated intrinsically by *E. coli*, was contained in the medium in addition to glucose (Fig. 4a). In contrast, the l-lysine production by the recombinant was increased when maltose, isomaltose, and panose were all contained in the medium. In the case of isomaltose utilization by the recombinant, l-lysine accumulation in the culture broth was increased by approximately 0.5 g/L; in contrast, in the case of panose, it was increased by approximately 0.2 g/L, showing lower utilization efficiency compared with that for maltose and isomaltose (Fig. 4a). Residual sugar analysis (Fig. 4b) indicated that about 98% of supplemented isomaltose and 90% of panose were consumed by the recombinant. Although small amounts of isomaltose and panose remained in the culture broth, the assimilation of isomaltose and panose was clearly enhanced by the introduction of *glvA* and *glvC.* These results showed that isomaltose and panose could be utilized as carbon sources and converted to l-lysine, suggesting that the heterologous expression of *glvA* and *glvC* could increase the efficiency of glucose feedstock utilization.

## Discussion

The PTS is composed of the phosphohistidine carrier protein (HPr), the enzyme I (EI) component, and the enzymes EIIA, EIIB, and EIIC. Although heterologous expression of *glvA* (encoding phospho-α-glucosidase) and *glvC* (encoding EIICB) conferred upon *E. coli* the ability to assimilate isomaltose, panose, and various sugars and sugar alcohols, the combination of GlvA and GlvC did not provide all the components needed to produce PTS activity, functioning only as an EIICB enzyme. HPr and EI are not specific to particular sugars, and EIIAs also do not have strict substrate selectivity for each sugar and EIICB. For example, EIIA^Glc^ can interact with glucose-PTS and trehalose-PTS (Postma et al. 1993). Pikis et al. reported that AglA, a homolog of GlvC, interacts with EIIA^Glc^, which is encoded by the endogenous *crr* gene in *E. coli* (Pikis et al. 2006). Therefore, GlvC is also likely to interact with EIIA^Glc^ of *E. coli.* We disrupted the *crr* gene and tested whether the heterologous expression of *glvA* and *glvC* allowed the mutant to assimilate isomaltose*.* The *crr* mutant harboring the *glvA* and *glvC* plasmids could not grow on M9 medium containing isomaltose as a sole carbon source (data not shown). Our results suggested that GlvC (a single-chain polypeptide of EIIB and EIIC) derived from the gram-positive bacterium *B. subtilis* could associate with EIIA^Glc^ of *E. coli*, similar to AglA of *K. pneumoniae*.

In industrial production of valuable compounds with *E. coli*, purified sugars are rarely used due to high cultivation cost, and hence, various sugar mixtures are used as carbon sources (Gokarn et al. 2014; Eiteman et al. 2008). However, assimilation of many sugars starts sequentially after consumption of glucose with lag phase, resulting in the extension of culture time and decrease of productivity (Eiteman et al. 2008; Aidelberg et al. 2014) due to carbon catabolite repression (Görke and Stülke 2008). In order to overcome this problem, several researchers have attempted to confer upon *E. coli* the ability to assimilate arabinose (Hernández-Montalvo et al. 2001), xylose (Dien et al. 2002; Hernández-Montalvo et al. 2001), and maltose (Tsujimoto et al. 2006) even in the presence of glucose. For example, Hernández-Montalvo used a mutant devoid of the phosphotransferase system to escape catabolite repression. In this study, we demonstrated that the heterologous expression of *glvA* and *glvC* under constitutive promoter allows *E. coli* to assimilate maltose and isomaltose in the presence of glucose*.* Surprisingly, the recombinant could also assimilate various other sugars and sugar alcohols, including several trisaccharides. This genetic engineering expanded the metabolizable sugars of *E. coli* and could increase product yield when using glucose feedstock. We demonstrated that an L-lysine-producing *E. coli* harboring *glvA* and *glvC* converted isomaltose and panose to l-lysine efficiently. This approach should increase the efficiency of industrial fermentation using *E. coli* and would facilitate full utilization of valuable carbohydrate resources.
